# Women's Holiday Fund

**Published:** 1923-07

**Authors:** A. F. London, Manuel J. Bidwell, J. Scott Lidgett, Helen A. Pownall

**Affiliations:** (Bp. of Miletopolis); (Chairman of Committee)


### Women's Holiday Fund.

SIR,—May we once more through the columns of your paper call attention to the valuable work that is being done by the Women's Holiday Fund on behalf of those whose lives are passed in dismal surroundings and weighed down by heavy responsibilities and anxieties?

It is not sufficiently realized how great are the demands made upon the strength and courage of the working woman, and that she is worn out in the never-ending struggle to maintain merely a decent standard of life. She is a stranger to any form of relaxation or recreation, and is seldom, if ever, free from the burden of household cares. As has been truly said: "She combines in one tired personality the careers of mother, wife, nurse, cook, housemaid, bargain-hunter, laundress and dressmaker." It is not surprising that under such a strain, rendered still more intolerable in many cases by overcrowding, the one on whom so much of the happiness and comfort of the home depend should break down in health or grow stunted in mind and soul. The joy and refreshment which a fortnight's holiday at the seaside or in the country bring to some of these over-tired women is better imagined than described. The striking remark by one woman on her return from her holiday that she was looking so well that her husband quite fell in love with her testifies to the excellent results achieved.

Is it too much to ask that all those who enjoy their own holidays regularly every year should send a donation, large or small, to enable the Society, which is in urgent need of help, to send away as many women as possible? The full cost of a fortnight's holiday is under £3. For a mother and baby, for whom a special Holiday Home is run at St. Leonards, the cost is about £3 10s. All applicants contribute something towards their holiday, being assessed according to their circumstances. Donations will be gratefully received and acknowledged by the Secretary, Women's Holiday Fund, 76 Denison House, 296 Vauxhall Bridge Road, S.W.1.

A. F. LONDON,

MANUEL J. BIDWELL
(Bp. of Miletopolis).

J. SCOTT LIDGETT.

HELEN A. POWNALL
(Chairman of Committee).

